# Indy knockdown in mice mimics elements of dietary restriction

**DOI:** 10.18632/aging.100365

**Published:** 2011-08-22

**Authors:** Gerald I. Shulman, Stephen L. Helfand

**Affiliations:** ^1^ Howard Hughes Medical Institute and the Departments of Internal Medicine and Cellular & Molecular Physiology Yale School of Medicine, New Haven, CT 06520, USA; ^2^ Department of Molecular Biology, Cell Biology and Biochemistry, Brown University, Providence, RI 02912, USA

A new mouse knock-out of the *Indy* gene sheds light on the mechanisms by which a reduction in *Indy* may lead to a healthier life. Mutations in the *Indy* gene (for *I'm not dead yet*) were first described in flies where they dramatically extend life span without a loss in fertility, physical activity, flight velocity or metabolic rate [1, 2]. The *Indy* gene codes for a high-affinity dicarboxylate/citrate plasma membrane transporter found most abundantly at the plasma membrane of adult fat body, oenocytes and midgut cells, the primary sites of intermediary metabolism in the fly [3]. In support of the hypothesis that the *Indy* mutation might be altering metabolism in a manner similar to dietary restriction (DR), it has been shown that DR down-regulates *Indy* in normal flies and that *Indy* long-lived flies share several phenotypes with long-lived DR flies, including decreased insulin-like signaling, lipid storage, weight gain, and resistance to starvation, and an increase in spontaneous physical activity [4].

In a new report in *Cell Metabolism* [5], knock-out of a mammalian homolog of *Indy* (mINDY; SLC13A5) shows dramatic effects in protecting mice from the adiposity and insulin resistance that develop with high-fat feeding or aging, and leads to phenotypes similar to those seen in DR. mINDY-deleted mice exhibit increased hepatic mitochondrial biogenesis, lipid oxidation, and energy expenditure, with a concomitant decrease in hepatic de novo lipogenesis and a reduction in ATP/ADP ratio, leading to activation of hepatic AMPK, induction of PGC-1alpha, inhibition of ACC-2, and reduction of SREBP-1c. These mice have reduced body weight, and preserve normal insulin signaling in the face of a high-fat diet or aging, demonstrating that some of the positive effects of *Indy* in flies can be extended to mammals. The profound beneficial effects on mammalian energy metabolism suggest that *Indy* is a potential target for treatment of obesity and type 2 diabetes. By focusing on the potential role of mammalian INDY in prolonging healthy life span, we stand to obtain new agents for extending healthy life span and to gain valuable insight into the genetic underpinnings of normal aging.
